# An Analysis of the Mycotoxins, Cytotoxicity, and Biodiversity of Airborne Molds Belonging to *Aspergillus* Genera Isolated from the Zoological Garden

**DOI:** 10.3390/pathogens14040332

**Published:** 2025-03-31

**Authors:** Kinga Plewa-Tutaj, Zuzanna Chmielewska, Magdalena Twarużek, Robert Kosicki, Ewelina Soszczyńska

**Affiliations:** 1 Department of Microbial Ecology and Acaroentomology, Faculty of Biological Sciences, University of Wrocław, 51-148 Wrocław, Poland; 325603@uwr.edu.pl; 2 Department of Physiology and Toxicology, Faculty of Biological Sciences, Kazimierz Wielki University, 85-064 Bydgoszcz, Poland; twarmag@ukw.edu.pl (M.T.); robkos@ukw.edu.pl (R.K.); eweso@ukw.edu.pl (E.S.)

**Keywords:** molds, *Aspergillus*, cytotoxicity, mycotoxins, zoo

## Abstract

The present study aimed to identify airborne molds of the *Aspergillus* genus and to determine the secondary metabolite profiles and toxicity of dominant fungal species isolated from various locations in the Wroclaw Zoological Garden. Air samples were collected using a MAS-100 air sampler and analyzed for fungal colony-forming units (CFU). Morphological and molecular methods, including ITS sequencing, were employed for dominant mold identification. The most frequently encountered species were *A. fumigatus* and *A. niger*, while *A. pseudoglaucus* and *A. nomius* were the least common. The high prevalence of species from sections *Nigri*, *Flavi*, and *Fumigati* suggests their adaptability to the zoo environment. A total of 17 *Aspergillus* isolates were analyzed for both their capacity to induce cellular toxicity and their production of mycotoxins. The results indicated that all isolates exhibited cellular toxicity, with 70.6% displaying levels of toxicity that were medium to high. Furthermore, the mycotoxicological analysis revealed that only *A. fumigatus* strains were capable of producing mycotoxins, specifically gliotoxin. The study underscores the discrepancy between the levels of toxicity and the production of mycotoxins, thereby suggesting the presence of additional cytotoxic metabolites. These findings emphasize the need for a comprehensive understanding of the complex interplay between fungal metabolites and their consequences for human health.

## 1. Introduction

Airborne fungal spores are a significant component of bioaerosols, impacting not only environmental quality but also human and animal health [1]. The most prevalent genera include *Cladosporium*, *Alternaria*, *Penicillium*, and *Aspergillus*, which encompass a variety of species known to thrive in diverse environments. However, in relation to zoological species, *Aspergillus* has been identified as a significant pathogen. [2] The nature of zoos as enclosed spaces with a variety of animal and plant life creates a special place where both animals and humans are exposed to potentially harmful microorganisms. The combination of animal and floral habitats with human visitors results in an ecosystem where *Aspergillus* spores can easily disseminate [3].

*Aspergillus* species are ubiquitous in the environment, thriving in soil, decaying vegetation, and air [4]. They are capable of producing vast quantities of spores, which when dispersed in the atmosphere, are readily inhaled by animals and humans alike [5]. In a zoo setting, this presents a dual threat: the health of the animals, particularly those with compromised immune systems, and the well-being of the staff and visitors. In the confined and controlled environment of a zoo, animals may be more susceptible to infections due to factors such as stress, captivity, and close contact with humans and other animals. *Aspergillus* has the potential to negatively impact human health due to its toxicity, allergenicity, and capacity to cause infection. Some *Aspergillus* species are known to produce secondary metabolites or mycotoxin [4]. Although the *Aspergillus* genus comprises several hundred species, only a limited number have been recognized as having a notable impact on human and animal health. Infections are most commonly associated with *A. flavus*, *A. fumigatus*, *A. nidulans*, *A. niger*, and *A. terreus*. Among these, *A. fumigatus* is frequently reported as a dominant pathogen, accounting for the majority of cases in numerous studies. However, the relative prevalence of specific *Aspergillus* species can vary significantly depending on geographical region and local environmental factors, with non-*fumigatus* pathogens being increasingly reported as causative agents in certain areas worldwide [5,6]. *A. fumigatus* is a particularly significant pathogen, known to cause a range of diseases, especially in individuals with compromised immune systems. It can lead to invasive aspergillosis, a severe infection that can spread from the lungs to other parts of the body, including the brain, heart, and kidneys [5]. This species is also known to produce mycotoxins, such as gliotoxin, which have been shown to suppress the immune system and exacerbate infections [1]. *A. flavus*, on the other hand, is an important pathogen known for the production of aflatoxins, potent carcinogens that contaminate food and pose serious health risks, while *A. niger* can cause respiratory infections and produce ochratoxin A, a mycotoxin that can damage the kidneys [5]. Additionally, fungi from the *Nigri* section are significant producers of fumonisin B2. Ochratoxin A is primarily produced by *A. carbonarius*, with *A. niger* contributing less frequently. In contrast, fumonisin B2 is predominantly associated with *A. niger*. These mycotoxins are known for their carcinogenic properties and pose substantial risks to human and animal health. Moreover, black aspergilli produce other secondary metabolites, such as malformins and bicoumarins, which may also have toxicological significance [7].

The presence of molds belonging to the genus *Aspergillus* in indoor environments, such as zoological gardens, necessitates comprehensive analysis and monitoring. Such analysis and monitoring is imperative to facilitate an understanding of the potential health implications of these molds, especially for both animals and humans, who are frequently exposed to such habitats. Several studies have emphasized the significance of indoor air quality in zoological facilities and the importance of regular environmental monitoring to identify and mitigate potential microbial hazards. Therefore, this study had two objectives: firstly, to identify the airborne molds belonging to the *Aspergillus* genus, and secondly, to analyze the secondary metabolite profiles and cytotoxicity of the predominant fungal species collected from the air in specific rooms of the Zoological Garden.

## 2. Materials and Methods

### 2.1. Study Area 

**Figure 1:**
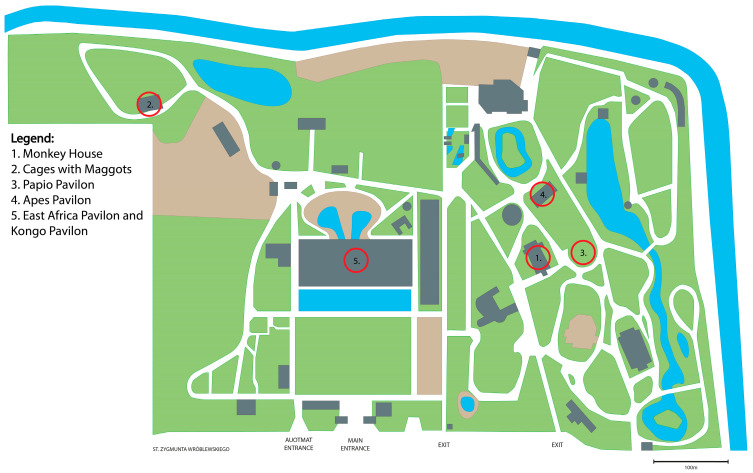
Locations of the sampling sites. The figure has been created from a map located at https://zoo.wroclaw.pl.

The research was carried out at the Zoological Garden in Wroclaw, distributed across twenty different facilities: the Monkey House (two sites), the Apes Pavilion (four sites), the Papio Pavilion (one site), the Maggots Pavilion (three sites), the Kongo Pavilion (five sites), and the East Africa Pavilion (five sites). In the Monkey House, sampling points were accessible to visitors without direct animal contact. The Apes Pavilion had visitor-accessible and staff-only sampling points, with the latter being clean. The Papio Pavilion had poor ventilation and was sampled before cleaning, leaving bedding material, food remnants, and excrement on the floor. Cages with maggots were sampled post-cleaning, resulting in no food remnants. However, the cages exhibited poor ventilation. The East Africa and Kongo Pavilions allowed direct visitor–animal contact. Ventilation was adequate, ensuring proper air circulation, but the environment remained highly humid with elevated temperatures. The selection of the study sites was based on their convenient accessibility. The locations of the sampling sites are shown in Figure 1.

### 2.2. Sampling Method 

Air samples were collected four times using a MAS-100 air sampler (Merck KGaA, Darmstadt, Germany). At the center of each sampling location, three simultaneous samples were taken: two were incubated at 27 °C, while the third was incubated at 37 °C, with all samples being deposited on Sabouraud agar surfaces. The airflow rate of the probe was set at 11 m/s, with a sampling frequency of 100 L/min. The total volume of air collected during the study was 20 L, which was determined through experimental means to be the maximum volume for mycological analysis in the area under study. Following a period of incubation, the number of fungal colonies was enumerated in terms of colony-forming units (CFU) per cubic meter of air (CFU/m^3^).

### 2.3. Characterization of Meteorological Conditions

Throughout each sampling procedure, the air temperature and relative humidity were monitored with the use of a thermo-hygrometer (model HI9565 HANNA, Warsaw, Poland). The average temperatures across the locations ranged from 15.5 °C to 24.8 °C, while humidity levels varied between 37.38% and 75.98%.

### 2.4. Morphological and Molecular Identification of Molds

All *Aspergillus* molds that were isolated were identified initially using diagnostic keys [8,9,10]. Subsequently, molecular methods were employed to identify the dominant isolated strains. The extraction of DNA was conducted using the Tissue DNA Purification Kit (EURx) following the manufacturer’s protocol. The molecular analyses were predominantly based on the sequence of the internal transcribed spacer ITS. The ITS region was amplified utilizing two primers: ITS1 (5′-TCCGTAGGTGAACCTGCGG-3′) and ITS4 (5′-TCCTCCGCTTATTGATATGC-3′). The PCR reactions were conducted in a T100 Thermal Cycler (Bio-Rad, Warsaw, Poland) with a total reaction volume of 12.5 µL, comprising 6.25 µL of 2× PCR Mix Plus (A&A Biotechnology, Gdańsk, Poland), 0.625 µL of each primer (10 mM), 4 µL of the DNA template, and 1 µL of deionized water (ddH_2_O). The thermal cycling conditions included an initial denaturation at 95 °C for 30 s, followed by 34 cycles of 95 °C for 45 s, 55 °C for 60 s, and 72 °C for 60 s. The process concluded with a final elongation step at 72 °C for 3 min. The PCR products were separated through electrophoresis on a 1% agarose gel pre-stained with SimplySafe (EURx, Gdańsk, Poland). Following this step, samples that yielded positive PCR results were purified and sequenced at Macrogen in Amsterdam, Netherlands, using the same primers that were applied during the DNA amplification process. The resulting nucleotide sequences were then manually edited with the DNA Baser Sequence Assembly software (4.0) from Heracle BioSoft SRL, Romania. The consensus sequences were then aligned and compared with those stored in the GenBank database of the National Center for Biotechnology Information (NCBI, Bethesda, MD, USA) using the BLAST algorithm (2.2.18).

### 2.5. MTT Test for Assessing Fungal Cytotoxicity

For the cytotoxicity assays, we utilized the quantitative colorimetric MTT test (3-(4,5-dimethylthiazol-2-yl)-2,5-diphenyltetrazolium bromide). This technique is frequently employed to assess the cytotoxic effects of various substances, including molds and their metabolites, bacterial cultures, and even food or feed samples. The MTT assay is based on the conversion of the yellow tetrazolium salt to the purple formazan, which is insoluble in water. This reaction occurs exclusively in living cells. The comprehensive methodology employed in this study is documented in the preceding article [11,12]. The swine kidney cells were maintained in Minimum Essential Medium Eagle (MEM; Sigma-Aldrich, St. Louis, MI, USA) enriched with an antibiotic solution containing penicillin (10,000 units/mL) and streptomycin (10 mg/mL) dissolved in 0.9% NaCl (Sigma-Aldrich), along with 5% fetal calf serum (Sigma-Aldrich). The culture process was carried out in a CO_2_ incubator (CB, BINDER GmbH, Tuttlingen, Germany) set at 5% CO_2_, 37 °C, and 98% humidity. The formation of formazan was measured by spectrophotometric absorbance using an ELISA microplate reader (ELISA LEDETECT 96, Biomed Dr. Wieser GmbH, Salzburg, Austria) at a wavelength of 510 nm (= the maximum absorption wavelength of formazan derivatives). The toxicity threshold was defined as the lowest concentration that caused a decrease in sample absorbance to values <50% of cell metabolic activity. The IC_50_ was determined from the absorbance result.

### 2.6. Sample Preparation and Mycotoxin Analysis

The sample preparation and mycotoxin analysis were carried out based on the methodology outlined by Plewa-Tutaj et al. (2024) [13]. To summarize, 2.0 g of the homogenized sample underwent vigorous shaking for 60 min with 8.0 mL of a solution composed of acetonitrile, water, and acetic acid in a 79:20:1 (*v*/*v*/*v*) ratio. The obtained mixture was subsequently centrifuged at 7000 rpm for 10 min. Following centrifugation, 40 µL of the supernatant was diluted in 960 µL of a methanol–water solution (2:8, *v*/*v*) and centrifuged again at 14,500 rpm for 30 min.

Mycotoxins were analyzed using high-performance liquid chromatography (HPLC) coupled with tandem mass spectrometry (MS/MS) detection. The HPLC system used was a Shimadzu Nexera (Kyoto, Japan), integrated with a 5500 QTrap mass spectrometry detector from Sciex (Foster City, CA, USA). Chromatographic separation was carried out on a Gemini C18 column (150 × 4.6 mm, 5 μm) from Phenomenex (Torrance, CA, USA). The method employed a flow rate of 1.0 mL/min and an injection volume of 5.0 μL. The mobile phase consisted of two distinct solvent mixtures: phase A, composed of methanol, water, and acetic acid in a 10:89:1 (*v*/*v*/*v*) ratio, and phase B, consisting of methanol, water, and acetic acid in a 97:2:1 (*v*/*v*/*v*) ratio. Both phases were supplemented with 5 mmol/L ammonium acetate to enhance ionization efficiency. The chromatographic gradient was programmed to maintain 0% phase B for the first 2.0 min, followed by a linear increase to 50% phase B between 2.0 and 5.0 min. Subsequently, the proportion of phase B was increased to 100% from 5.0 to 14.0 min, and maintained at this level until 18.0 min. At 18.0 min, the proportion of phase B was returned to 0%, where it was maintained until 22.5 min.

The quantification of mycotoxins was achieved using external calibration. The limits of detection (LOD) and quantitation (LOQ) for each mycotoxin were established by spiking a blank sample extract with known concentrations of mycotoxin standards. The LOD and LOQ values were determined based on signal-to-noise (S/N) ratios of 3:1 and 10:1, respectively, using mass spectrometry software. Detailed analytical parameters and validation data are provided in the study by Plewa-Tutaj et al. (2024) [13].

Using this method, each fungal isolate was tested for the presence of 37 diverse mycotoxins and secondary metabolites. These included nivalenol, deoxynivalenol, monoacetoxyscirpenol, diacetoxyscirpenol, T-2 toxin, HT-2 toxin, aflatoxins (M1, B1, B2, G1, and G2), patulin, fusarenon X, T-2 tetraol, α-zearalanol, β-zearalanol, α-zearalenol, β-zearalenol, 15-acetyldeoxynivalenol, 3-acetyldeoxynivalenol, deepoxy-deoxynivalenol, fumonisins (B1, B2, and B3), zearalenone, zearalanone, T-2 triol, griseofulvin, moniliformin, mycophenolic acid, neosolaniol, ochratoxins (A, B), roquefortine C, sterigmatocystin, gliotoxin, and mevinolin.

## 3. Results and Discussion

### 3.1. Concentration Levels of Airborne Molds

**Figure 2:**
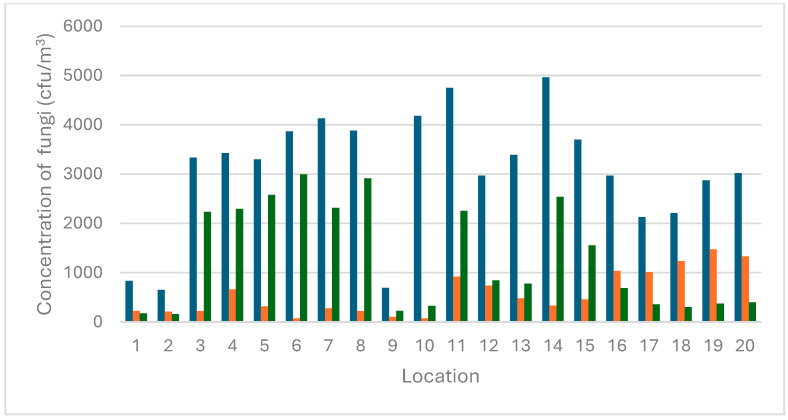
The air fungal concentration (CFU/m^3^) in 20 different locations at a zoological garden. Legend: 1–2 (Monkey House), 3–5 (cages with maggots 1), 6–9 (Apes Pavilion), 10 (Papio Pavilion), 11–15 (Kongo Pavilion), and 16–20 (East Africa Pavilion). Legend: dark blue bars (total cfu/m^3^), orange bars (cfu/m^3^ of *Aspergillus*), green bars (cfu/m^3^ of *Penicillium*).

The mycological contamination levels were measured across several locations, including the Monkey House, cages with maggots, and the Apes Pavilion, Papio Pavilion, Kongo Pavilion, and East Africa Pavilion. The average fungal counts, along with specific counts for *Aspergillus*, *Penicillium*, and other fungi, are summarized in Table 1 and Figure 2.

The Kongo Pavilion exhibited the highest total contamination level at 4963 cfu/m^3^ (Location 14), with *Aspergillus* at 331 cfu/m^3^, *Penicillium* at 2538 cfu/m^3^, and other fungi at 2094 cfu/m^3^. Conversely, the Monkey House exhibited the lowest total contamination level at 650 cfu/m^3^ (Location 2), with *Aspergillus* at 211 cfu/m^3^, *Penicillium* at 162 cfu/m^3^, and other fungi at 275 cfu/m^3^. These findings suggest that the environmental conditions in the Monkey House are less conducive to fungal growth compared to other locations. The East Africa Pavilion exhibited the highest *Aspergillus* contamination levels at 1475 cfu/m^3^ (Location 19), while the Apes Pavilion and Papio Pavilion demonstrated the lowest *Aspergillus* contamination levels at 75 cfu/m^3^ (Location 6 and Location 10). *Penicillium* contamination levels were observed to be the highest in the Apes Pavilion at 2994 cfu/m^3^ (Location 6) and lowest in the Monkey House at 162 cfu/m^3^ (Location 2). The Papio Pavilion exhibited the highest contamination from other fungi at 3781 cfu/m^3^ (Location 10), while the Monkey House showed the lowest at 275 cfu/m^3^ (Location 2).

A recent analysis has revealed significant relationships between environmental factors and the prevalence of *Aspergillus* fungi in various locations within a zoological garden. The study found a strong positive correlation (0.74) between temperature and the number of these fungi, indicating that higher temperatures are associated with an increased count of *Aspergillus.* This suggests that temperature is a critical factor influencing the growth and distribution of these fungi. In contrast, the correlation between humidity and the *Aspergillus* count was found to be weaker (0.21). While there was a slight tendency for a higher humidity to be associated with higher counts of fungi, the impact of humidity was less significant compared to temperature. This indicates that other factors play a more dominant role in determining the fungal counts. Further research is required to investigate the specific factors contributing to the observed variations in fungal contamination levels across different locations. It could be noticed that cfu/m^3^ in the Monkey House (Location 1 and 2) and in the Apes Pavilion (Location 9) is significantly lower compared to all other sampling sites. These areas are open and well ventilated, accessible to visitors, and without direct contact with animals or organic materials. In contrast, the Papio Pavilion (Location 10) exhibited one of the highest values of cfu/m^3^. This location was confined, poorly ventilated, and had an accumulation of organic material. It might suggest that restricted airflow and the presence of decomposing organic matter may contribute to the increasing proliferation and aerosolization of fungal spores. Furthermore, it highlights the critical role of good ventilation and environmental hygiene in enclosed spaces intended for keeping animals in good health and condition. 

The assessment of mycological pollution levels in zoological gardens is a relatively underexplored area, with limited studies available in the scientific literature. This paucity of data poses significant challenges for a comprehensive analysis and comparison. Research conducted in the Silesian Zoological Garden in Chorzów, Poland, revealed significant variations in airborne fungal levels depending on the type of animals and the microclimatic conditions. For example, Grzyb and Boroń (2020) found that the lowest total fungal aerosol concentration was recorded in the hippo enclosures, measuring approximately 970 CFU/m^3^ [14]. In contrast, the highest concentration was observed in the exotarium, reaching 16,800 CFU/m^3^ [14]. The study indicated that while the overall concentration of airborne fungi was below recommended limits, there were notable variations across different enclosures. It has been demonstrated that analogous relationships have been exhibited in the author’s own research. In addition, other studies carried out at the Wrocław Zoo have found that fungal concentrations in the air ranged from 50 to 36,500 CFU/m^3^, consistently remaining below the recommended threshold of 50,000 CFU/m^3^ for fungi. The analysis indicated that environmental factors, specifically temperature and relative humidity, exerted a significant influence on the presence and concentration of airborne fungi. These studies, which are pioneering works in the field of mycological contamination in zoos, highlight the lack of comprehensive data on the subject. As such, they provide an important foundation for understanding the dynamics of microbial contamination in zoos. However, they also highlight the need for further, more comprehensive investigations to fully elucidate the patterns and implications of such contamination for animals and workers.

### 3.2. The Biodiversity of Airborne Fungi Belonging to Aspergillus Genera

In this study, various *Aspergillus* species were identified in different locations within a zoological garden using both molecular and morphological methods. The identified species included *A. ochraceus, A. flavus*, *A. fumigatus, A. steynii, A. tamarri, A. pseudoglaucus, A. niger, A. nomius, A. westerdijikiae, A. ostianus, A. tubingensis, A. sydowii, A. elegans*, and species belonging to sections *Nigri, Circumdati, Fumigati, Flavi*, *and Nidulantes* (Table 2).

The combination of these methods enabled the accurate identification of a wide range of *Aspergillus* species, highlighting their ecological adaptability and significance within the zoological garden. *A. fumigatus* and *A. niger* were among the most frequently encountered species, reflecting their known resilience and ability to thrive in diverse environments. In contrast, *A. pseudoglaucus* and *A. nomius* were the least frequently occurring species, each found in only one location. The high prevalence of species from the *Aspergillus* sections *Nigri, Flavi*, and *Fumigati* suggests that these groups are particularly well suited to the conditions found in zoological gardens. The presence of these fungi in different locations within the zoological garden raises important questions about their ecological roles, potential interactions with animal hosts, and implications for animal health. These fungi are known for their pathogenic potential and ability to produce harmful mycotoxins. *A. fumigatus*, for example, can produce gliotoxin, which suppresses immune function, increasing susceptibility to respiratory infections (aspergillosis) in animals and humans [15]. *A. flavus* is distinguished by its capacity to produce aflatoxins, which are potent carcinogenic substances that have the potential to contaminate animal feed, resulting in liver damage and immune suppression in animals, and posing serious health risks to humans. *A. niger* produces ochratoxin A, a substance that can cause kidney damage and pose a risk of respiratory infections and mycotoxicosis in animals, and can cause allergic reactions and respiratory issues in humans [16,17]. The results highlight the importance of implementing robust monitoring and control strategies to reduce the health hazards linked to *Aspergillus* contamination in zoological settings.

In our previous study, we identified several fungal species, with *Penicillium*, *Aspergillus*, and *Cladosporium* being the most prevalent genera. *Penicillium* species dominated the fungal population, accounting for 58.9% of the total fungal strains, followed by *Aspergillus* at 25.89%, and *Cladosporium* at 3.57%. The study also identified other genera, including *Talaromyces*, *Mucor*, *Schizophyllum*, *Syncephalastrum*, *Alternaria*, *Absidia*, and *Cunninghamella*, which collectively contribute to the overall biodiversity of the fungal community (Plewa-Tutaj et al., 2024) [13]. However, other studies have highlighted that *Aspergillus* is a significant pathogen among zoological species. These studies emphasize that *Aspergillus* spores levels are significantly higher in enclosed spaces lacking HEPA filtration, increasing the infection risk for both animals and humans [2]. Another research conducted in a colony of Humboldt penguins at a Parisian zoo found that the burden of *Aspergillus* spores in penguin nests significantly rises with increasing outdoor temperatures [18].

### 3.3. Analysis of Cytotoxicity and Mycotoxin Production

Seventeen isolates belonging to the *Aspergillus* genera (sections *Fumigati* and *Flavi*), which are the two most frequently observed sections, were selected for cytotoxicity and mycotoxicological analyses. Table 3 presents the MTT cytotoxicity test results for these strains. All *Aspergillus* isolates tested were found to be cytotoxic. Specifically, eleven strains (70.6%) exhibited medium to high levels of cytotoxicity, while five strains demonstrated low cytotoxicity. The strains with the highest levels of cytotoxicity belonged to the *Aspergillus* section *Fumigati* (1AJ, 5AJ, 5CJ, 8W, and 19L).

The area of research concerning the health implications of airborne mycotoxins remains underexplored, with the majority of studies thus far concentrating on the assessment of cellular toxicity through the utilization of established cell line assays, such as the MTT test. The extant literature principally addresses the toxicity of fungi in domestic environments, hospital wards, museums, composting plants, and tanneries, with a significant focus on *Aspergillus* species [19,20,21]. However, there remains a scarcity of research on mycotoxins in zoological gardens and other establishments associated with animal husbandry. A previous study was conducted in a zoological garden on 83 mold strains, comprising 52 *Penicillium* and 32 *Aspergillus* isolates. The MTT assay was also employed to assess the viability of the strains, and the results indicated that 97.6% of the analyzed strains exhibited toxicity. Specifically, 98.07% of *Penicillium* strains demonstrated toxicity, with 55.7% exhibiting medium to high levels of toxicity, and a similar proportion (93.75%) of *Aspergillus* strains were cytotoxic, with 71.88% exhibiting medium to high toxicity. The most cytotoxic strains included *A. westerdijkiae*, *A. ostianus*, *A. giganteus*, *A. fumigatus*, *P. griseofulvum*, *P. chrysogenum*, *P. citrinum, P. steckii,* and *P. sumatraense*. These preliminary studies provide only initial insights into the mold species found in zoo environments, and thus in the potential risk of airborne fungal mycotoxins [13]. The limited data on the toxicity of molds isolated from these settings hinders the possibility of a broader comparative analysis. Given the current lack of data, it is not yet possible to discuss occupational exposure. Further research is required to fully understand the impact of mycotoxins and molds in occupational environments.

In the course of the mycotoxicological analysis of 17 fungal strains, it was found that only eight strains belonging to the genus *A. fumigatus* were capable of producing a single mycotoxin, gliotoxin. The present study identified this toxin as the only mycotoxin detected. Conversely, the concentrations of other toxins and secondary metabolites for the other fungal strains were found to be below the limit of detection (LOD). However, it is probable that *A. fumigatus,* as well as other species, can produce other mycotoxins under different conditions, in alternative media, or with lower limits of detection (LOD). 

Gliotoxin is a sulfur-containing mycotoxin, classified as an epidithiodioxopiperazine (ETP), and is known for its significant role in the pathogenicity of *A. fumigatus*. Furthermore, the inhalation of gliotoxin poses serious health risks due to its potent immunosuppressive and cytotoxic effects, which can result in weakened immune responses, chronic infections, and respiratory issues in both humans and animals [22]. The remaining strains that were examined in the experiment did not demonstrate any capacity to generate mycotoxins. Another survey conducted by our team demonstrated analogous outcomes for strains of the genus *Aspergillus*, wherein 23 (71.88%) of the 32 *Aspergillus* isolates that were examined for their capacity to produce mycotoxins exhibited positive results. Of the nine *A. fumigatus* strains examined, eight were found to produce gliotoxin, and three strains were found to produce roquefortin C [13].

In the present study, the majority of fungal strains exhibited toxicity when tested with the MTT assay. However, only a small proportion of these strains produced detectable levels of mycotoxins. This discrepancy between toxicity and mycotoxin production can be attributed to several factors. Firstly, the MTT assay measures cell viability based on the metabolic activity of living cells. This means that any metabolites produced by the fungi, not just mycotoxins, can affect the outcome of the test. Secondly, fungi produce a wide array of secondary metabolites, some of which may possess cytotoxic properties independent of their mycotoxin content [23]. These metabolites can interfere with cellular respiration and mitochondrial function, leading to the reduced cell viability observed in the MTT assay. Thirdly, fungal strains possess varied metabolic pathways that can lead to the production of different toxic compounds. It is noteworthy that some fungi may produce cytotoxic metabolites that do not fall under the classification of mycotoxins; nevertheless, they can contribute to the overall toxicity of the sample. The presence of these non-mycotoxin metabolites can result in cytotoxic effects in the MTT assay, even in the absence of significant mycotoxin production [24]. Furthermore, the interaction between different metabolites, including mycotoxins, has been demonstrated to result in synergistic effects that amplify the cytotoxic potential of the compounds in question. In the event of multiple toxic compounds being present, it has been shown that they may work together to enhance cellular damage, leading to greater cytotoxic effects than would be expected from individual compounds alone [25]. In addition, environmental conditions, the growth medium, and strain-specific genetic factors have been shown to influence the production of cytotoxic metabolites. Variability in these factors can lead to differences in the metabolic profiles of the fungi, resulting in varying levels of observed toxicity in the MTT assay [26]. The observed toxicity in the majority of fungal strains tested using the MTT assay can be attributed to the production of a diverse range of cytotoxic metabolites, not limited to mycotoxins. It is therefore vital to understand the complex interactions between these metabolites and their impact on cell viability if an accurate assessment of the cytotoxic potential of fungal strains is to be made.

## 4. Conclusions

Mycological surveys in zoos are relatively rare compared to those conducted in other large-scale animal breeding facilities, highlighting the need for a greater attention to fungi present in such environments. This study provides valuable insights into the biodiversity of *Aspergillus* fungi, particularly their toxicity. The identification of various *Aspergillus* species, including the dominant *A. fumigatus*, *A. flavus*, and *A. niger*, demonstrates their resilience and adaptability across diverse environments. The study underscores the importance of a thorough monitoring and control of *Aspergillus* contamination in various settings to effectively mitigate the risks posed by their mycotoxin-induced toxicity. Our findings highlight the significant cytotoxic potential of isolates from the *Fumigati* section, as well as their ability to produce gliotoxin—a mycotoxin with well-established pathogenic implications. The absence of other detectable mycotoxins in our study suggests the necessity for further research, not only under varying environmental conditions but also using alternative media and applying detection methods with lower limits of detection (LOD) to gain a more comprehensive understanding of the mycotoxin-producing capacities of *Aspergillus* species.

A detailed understanding of the factors affecting these fungi, such as humidity, temperature, and the availability of organic matter, is crucial for developing effective strategies to mitigate their impact. This knowledge can help develop better environmental controls and improve health and safety measures. Moreover, there is a need for more extensive data from zoos to achieve a full understanding of the ecological roles and health effects of these fungi on both animals and humans. 

## Figures and Tables

**Table 1:** The average air fungal concentration (CFU/m^3^) in 20 different locations at a zoological garden.

Location	Average T (°C)	Average H (%)	Total cfu/m^3^	cfu/m^3^ *Aspergillus*	cfu/m^3^ *Penicillium*	cfu/m^3^ Different Species
1	22.13	41.28	837	225	175	437
2	21.83	37.38	650	211	162	275
3	16.48	68.05	3335	221	2236	878
4	17.13	70.45	3425	662	2295	468
5	16.83	67.05	3300	319	2581	400
6	19.35	56.43	3869	75	2994	800
7	17.38	51.78	4134	275	2318	1550
8	18.65	64.03	3886	221	2914	750
9	19.88	42.60	694	106	225	362
10	15.5	75.98	4181	75	325	3781
11	21.16	61.50	4750	922	2257	1571
12	20.80	62.25	2972	736	843	1393
13	45798	63.63	3393	479	779	2137
14	20.93	61.65	4963	331	2538	2094
15	21.13	60.80	3700	457	1557	1686
16	22.53	70.93	2969	1037	687	1243
17	24.38	58.80	2128	1014	357	757
18	24.60	59.78	2212	1237	300	675
19	24.80	58.28	2875	1475	375	1025
20	24.62	61.38	3020	1330	400	1290

Legend: 1–2 (Monkey House), 3–5 (cages with maggots 1), 6–9 (Apes Pavilion), 10 (Papio Pavilion), 11–15 (Kongo Pavilion), and 16–20 (East Africa Pavilion).

**Table 2:** The biodiversity of *Aspergillus* species/section.

No	Species	Zoological Garden																			
		1	2	3	4	5	6	7	8	9	10	11	12	13	14	15	16	17	18	19	20
1	*A.* *ochraceus*	+	+	+						+				+	+						
2	*A.* *flavus*		+						+		+					+					
3	*A.* *fumigatus*			+	+		+				+			+	+	+					
4	*A.* *steynii*					+				+	+	+									
5	*A.* *tamarri*					+						+	+								
6	*A.* *pseudoglaucus*					+															
7	*A.* *niger*							+				+			+		+	+	+		+
8	*A.* *nomius*																			+	
9	*A.* *westerdijikiae*											+	+		+	+					
10	*A.* *ostianus*											+		+				+		+	
11	*A.* *tubingensis*														+	+	+				
12	*A. sydowii*									+							+				
13	*A.* *elegans*																	+			+
14	*Aspergillus* section *Nigri*	+	+				+	+	+		+	+	+	+	+	+	+	+	+		+
15	*Aspergillus* section *Circumdati*	+		+		+				+				+	+	+	+	+	+	+	
16	*Aspergillus* section Fumigati	+	+	+	+	+	+	+	+	+	+	+	+	+	+	+	+	+	+	+	+
17	*Aspergillus* section *Flavi*		+			+			+	+	+	+	+	+		+	+	+	+	+	+
18	*Aspergillus* section *Nidulantes*									+											

Legend: The presence of the plus sign (+) indicates the identification of a species at a given location.

**Table 3:** An analysis of airborne molds belonging to *Aspergillus* genera using the MTT test.

Species	IC_50_ [cm^2^/mL]
Section *Flavi* 4cz	3.64
Section *Flavi* 5bz	3.9
Section *Flavi* 14bz	5.67
*A. flavus* 15w	2.07
*A. fumigatus* 1aj	0.09
*A. fumigatus* 5aj	0.17
*A. fumigatus* 5cj	0.17
*A. fumigatus* 7l	0.3
*A. fumigatus* 8w	0.21
*A. fumigatus* 9cl	0.36
*A. fumigatus* 10z	4.37
*A. fumigatus* 19l	0.14
*A. fumigatus* 26l	0.41
*A. tamarii* 1l	18.36
*A. tamarii* 14al	10.24
*A. tamarii* 6w	9.73
*A. tamarii* 12z	8.77

## Data Availability

The datasets generated during and/or analyzed during the current study are available from the corresponding author on reasonable request.
